# Brain Stimulation for Combating Alzheimer’s Disease

**DOI:** 10.3389/fneur.2014.00080

**Published:** 2014-06-02

**Authors:** Niels Hansen

**Affiliations:** ^1^Department of Neurophysiology, Medical Faculty, Ruhr University Bochum, Bochum, Germany

**Keywords:** Alzheimer’s disease, brain stimulation, cognition, memory, dorsolateral prefrontal cortex, transcranial magnetic stimulation, transcranial direct current stimulation, deep-brain stimulation

Alzheimer’s disease (AD) is a devastating disease affecting 5.2 million Americans. As the cause of death between 2000 and 2010, AD increased by 68% (1). The number of individuals developing AD in the United States will rise dramatically in the following decades (2). As AD patients are often resistant to pharmacotherapy, alternative therapeutic strategies are imperative. Non-invasive and non-lesional brain stimulation is a promising therapeutic option that has been attracting increasing attention over the last few years (3–6). Brain stimulation is useful to accelerate diagnosis and treatment (6, 7). This article focuses on advances in cognitive neurorehabilitation via brain stimulation techniques in AD patients to provide insights into a promising ray of hope for AD patients.

## Non-Invasive Brain Stimulation

### Transcranial magnetic stimulation

Transcranial magnetic stimulation (TMS) modulates cortical activity non-invasively (4). Repetitive transcranial magnetic stimulation (rTMS) creates magnetic pulses to the scalp delivered through a coil at a rhythmic repetition rate. The magnetic pulse causes cortical neurons to depolarize (8). TMS is an important cortical stimulation method for the adjunctive treatment of neurodegenerative disorders such as Parkinson’s disease (9). Furthermore, TMS can improve cognitive function in neuropsychiatric disorders (10). RTMS studies revealed the pivotal role of the prefrontal cortex (PFC) during information encoding and retrieval (11–15). Furthermore, as neuroimaging studies revealed, heightened activity in the dorsolateral PFC (DLPFC) is one of the brain abnormalities associated with AD (16, 17). These changes in brain activity in the DLPFC underpin the recruitment of compensatory networks (18, 19). It would thus make sense to modulate the PFC’s neural activity to modify memory function, the most prominent feature of disturbed cognition in AD. There is solid evidence that high-frequency rTMS over the DLPFC is superior to low-frequency rTMS in treating cognitive dysfunction in AD patients as measured by the mini mental state examination (MMSE) (20). The first studies using TMS in AD showed that high-frequency rTMS of the DLPFC improves naming accuracy. Demented patients often display impaired naming ability (21). RTMS improved both action and object naming in a group of advanced AD patients (22, 23). Auditory verbal comprehension of continuous daily DLPFC–rTMS over 4 months was increased for up to 2 months after stimulation (24). As the inferior PFC plays a role in controlling memory (25), stimulating that part of the PFC in AD patients is a reasonable approach. Indeed, stimulation of the left inferior PFC resulted in enhanced episodic memory function (26). Alongside the PFC, the parietal cortices are important for information retrieval (27). RTMS of the parietal cortex advances the associative memory capacity in patients with mild cognitive impairment (MCI) (15). The combination of cognitive training with rTMS seems to benefit cognitive functions as much as treatment with cholinesterase inhibitors (28, 29). Moreover, TMS is useful for identifying early AD patients with cholinergic degeneration (30), and for monitoring the drug response (7). The biomarker of central cholinergic activity such as short-latency afferent inhibition (SAI) assessed by TMS is relevant to the drug response (31). Other TMS measures such as long-interval intracortical inhibition (LICI) are also worth considering for measuring drugs. Patients undergoing monotherapy or combination therapy with acetylcholinesterase inhibitors demonstrated impaired LICI when compared to healthy controls (7). Remarkably, the LICI values correlated with Alzheimer’s Disease Assessment Scale–Cognitive Subscale (ADAS–Cog) scores. These findings indicate that these neurophysiologic TMS parameters help us measure the response to anti-dementia drugs (7).

### Transcranial direct current stimulation

Transcranial direct current stimulation is a non-invasive tool to modulate cortical excitability via brain polarization with weak direct currents (3), and it is attracting greater attention in AD as a reinforcer of cognitive function (6). tDCS showed already promising results for its beneficial usage in both neurodegenerative and neuropsychiatric disorders (9, 10). The direct current affects the resting membrane potential and thereby the neuronal firing rate. The current’s polarity determines the excitability of cortical neurons: anodal tDCS (atDCS) increases whereas cathodal tDCS (ctDCS) lowers it (3). AtDCS has usually been shown to rectify visual and word recognition memory and working memory in AD patients when applied over the temporal cortex and DLPFC (32–34). The effect of temporal cortex atDCS persisted up to 1 month after therapy (35). AtDCS of the DLPFC can alter connectivity during the resting state (36). This might have diagnostic value, as AD patients’ resting-state brain electroencephalographic rhythms differ from those in control subjects (37). However, despite the obvious advantages of TMS and tDCS, both are limited to stimulating large surface cortical structures, so that the hippocampus and mediotemporal lobe structures are not accessed directly.

### Transcutaneous electrical nerve stimulation

On the contrary, transcutaneous electrical nerve stimulation (TENS) is believed to stimulate the hippocampus relevant to memory formation and the forebrain system degenerated in AD (38). TENS entails current applied transcutaneously to excite nerves, enhancing cognition in AD patients (38, 39). The hippocampus is stimulated by TENS via spinoseptal and brainstem nuclei such as the locus coeruleus (LC) and dorsal raphe nucleus (DRN) (38). The cholinergic basal forebrain system is reached by the LC and DRN via noradrenergic and serotonergic projections. TENS can induce noradrenergic and serotonergic neuromodulation by this means. There is ongoing debate as to whether the effect of TENS is more prominent in mildly or severely affected AD patients (38, 39). TENS is effective in improving visual memory, long-term face recognition memory, and word fluency in AD patients (40).

### Vagus nerve stimulation

Vagal nerve (VN) afferents reach the nucleus of the solitary tract (NST), and the LC is downstream to the NST. The VN’s influence on LC neurons is demonstrated by the fact that VN stimulation (VNS) induces a significant noradrenaline increase in the rat’s hippocampus (41). VNS improves cognitive function as measured by the ADAS–cog and MMSE in AD patients (42), AD patients demonstrated improvement or their cognitive function did not decline even a year after VNS according to the ADAS–cog and MMSE (43).

### Radio electric asymmetric and cerebellar theta burst brain stimulation

Radio electric asymmetric and cerebellar theta burst stimulation are two novel methods. Non-invasive radio electric asymmetric brain stimulation (REAC) uses frequency ranges of 2–11 Hz and consists of intermittent radio-frequency bursts lasting 500 ms (44). REAC enhances cognitive functions in AD patients according to different scales [MMSE, neuropsychiatric inventory (NPI), activities of daily living (ADL), and instrumental activities of daily living (IADL)] (44). A recent study revealed that cerebellar theta burst stimulation can restore cholinergic dysfunction in AD patients (45). They also showed that cerebellum stimulation might be a useful tool to improve cholinergic dysfunction in AD via the cerebello-thalamo-cortical pathway (45) so relevant to cognitive control (46).

## Invasive Brain Stimulation

### Deep-brain stimulation

Deep-brain stimulation (DBS) consists of administering rectangular current pulses into target brain structures [for review, see Ref. (5)]. The stimulation electrodes are implanted chronically. DBS is an established therapeutic option in Parkinson’s disease, dystonia, and tremor (47, 48). DBS has evolved to be one of the most effective treatments in Parkinson disease (49). Considering the increasing success of this technology in modulating activity in dysfunctional motor pathways, DBS is also attracting growing interest for modulating the activity in dysfunctional neural circuits in AD (5). An advantage of DBS is that memory structures can be assessed directly, unlike non-invasive brain stimulation. Bilateral DBS of the hypothalamus and fornix has led to improved recollection in memory functions (50), whereas high-frequency DBS of the fornix was clinically ineffective despite the observation of increased metabolic activity in temporal lobe structures (51). Fornix–DBS stabilized memory function in AD patients in tests such as the MMSE, ADAS–Cog, Free and Cued Selective Reminding Test (52). High resolution positron emission tomography studies revealed a persistent fornix–DBS effect on cerebral metabolism in memory processing structures 1 year after stimulation that correlated with improved cognitive and memory functions (53). DBS of the entorhinal cortex can induce phase resetting of hippocampal theta oscillations in humans (54). Theta resetting can enhance the encoding of new information and enhance memory (55). DBS of the entorhinal area thus seems to be a promising target in treating pathological AD to enhance memory functions. DBS probably reduces memory dysfunction by promoting the physiological conditions and patterns of extracellular field potentials necessary for long-term memory (56). Furthermore, there is evidence in rodents that fornix and perforant path stimulation increases hippocampal neurogenesis and long-term potentiation to facilitate memory storage (5, 57, 58). The nucleus basalis of Meynert (NBM) has several cholinergic projections, and it degenerates in AD, thus the NBM is a budding future target for DBS in AD (5, 59). Another auspicious, but not yet investigated target of DBS in AD patients may be stimulation of the anterior thalamic nucleus, as prior to encoding, its stimulation improved verbal memory in epileptic patients (60). Whether bilateral or unilateral stimulation is more effective to enhance memory remains unresolved (50, 54). Moreover, the precise timing of DBS seems to be a key factor, as neurorehabilitation studies (61) have suggested that therapeutic intervention is most beneficial when applied during the learning or recall phase.

### Contrasting juxtaposition of the stimulation techniques

Taken together, TMS is the most frequently investigated and powerful non-invasive brain stimulation technique in AD patients on the basis of studies with different stimulation sites (Table 1). The DLPFC is the most evaluated stimulation target in AD patients for TMS (Table 1). tDCS and TMS offer the advantage of a non-invasive treatment and long-lasting effect. tDCS is less investigated than TMS in AD patients (Table 1). In my opinion, VNS and TENS represent also valuable, but less examined techniques that may be relevant to treating AD patients when TMS and tDCS are ineffective. Novel techniques such as REAC and cerebellar theta burst stimulation require more investigation to assess their efficacy in AD patients. However, these non-invasive techniques cannot be applied directly to structures involved in AD pathophysiology such as the NBM and hippocampus. DBS constitutes a valuable method for this purpose. DBS of the fornix and entorhinal area enables the modulation of memory functions. Due to its invasiveness, DBS may eventually be the *ultima ratio* in clinical settings if non-invasive stimulation such as TMS has not proven effective. However, given the therapeutic success of DBS in Parkinson’s disease, DBS in AD is also likely to become an upcoming alternative to pharmacotherapy. In the future, individual patient characteristics with risks and potential comorbidity profiles will have to be analyzed to determine the optimal stimulation technique for that patient.

**Table 1:** **Brain stimulation in Alzheimer’s disease patients: stimulation sites and clinical effects**.

Stimulation site	Technique	Clinical effect	Reference
Cerebellum	TBS	Cholinergic dysfunction ↓	(45)
DLPFC	TMS	MMSE ↑	(20)
	TMS	Naming accuracy ↑	(22, 23)
	TMS	Auditory verbal comprehension ↑	(24)
	tDCS	Working memory ↑	(32)
	tDCS	Declarative memory ↑	(62)
Ear	REAC	MMSE, NPI, (I)ADL ↑	(44)
Fornix	DBS	MMSE, ADAS–Cog, FCSR Test ↑	(52)
	DBS	Increased cerebral glucose metabolism, memory ↑	(53)
Hypothalamus and fornix	DBS	Memory recollection ↑	(50)
Inferior PFC	TMS	Memory ↑	(26)
Parietal cortex	TMS	Associative memory ↑	(15)
Spine (Th1–Th5)	TENS	Visual memory ↑	(40)
		Face recognition memory ↑	
Temporal cortex	tDCS	Visual recognition memory ↑	(33, 35)
Temporoparietal area	tDCS	Recognition memory ↑	(34)
Vagus nerve	VNS	ADAS–cog and MMSE ↑	(43)

ADAS–cog, Alzheimer’s Disease Assessment Scale–cognitive subscale; DBS, deep-brain stimulation; DLPFC, dorsolateral prefrontal cortex; FCSR, Free and Cued Selective Reminding Test; (I) ADL, instrumental activities of daily living; MMSE, Mini Mental State Examination; NPI, neuropsychiatric inventory; PFC, prefrontal cortex; REAC, radio electric asymmetric brain stimulation; TBS, theta burst stimulation; tDCS, transcranial direct current stimulation; TENS, transcutaneous nerve stimulation; TMS, transcranial magnetic stimulation; VNS, vagus nerve stimulation; ↑, beneficial effect; ↓, no beneficial effect.

## Conclusion

Having analyzed results from different techniques and stimulation sites, I believe that TMS, tDCS, and DBS are the brain stimulation methods with the brightest prospects in AD patients. Increased neural activity, connectivity, and synaptic plasticity in memory and cognition-related brain areas are potential mechanisms of action. Further intensive investigation is needed to implement stimulation protocols and targets in AD patients. The optimal stimulation therapy will have to be considered in accordance with individual patients’ health predisposition, risks, and other factors.

## Conflict of Interest Statement

The author declares that the research was conducted in the absence of any commercial or financial relationships that could be construed as a potential conflict of interest.
